# Increased exacerbations and hospitalizations among PI*MZ compared to PI*MM individuals: an electronic health record analysis

**DOI:** 10.1186/s12931-025-03322-6

**Published:** 2025-07-11

**Authors:** Vickram Tejwani, Yifan Wang, Lauren Munoz Tremblay, Elizabeth Azzato, Arianne K. Baldomero, Christine Wendt, Amy Attaway, Russell Bowler, Umur Hatipoglu, Rebecca Hutton, Charlie Strange, Xiaofeng Wang, Victor E. Ortega, Joe Zein, James K. Stoller

**Affiliations:** 1 Department of Pulmonary and Critical Care Medicine, Integrated Hospital Care Institute, Cleveland Clinic, Cleveland, USA; 2 Genomic Sciences and Systems Biology, Lerner Research Institute, Cleveland Clinic, Cleveland, USA; 3 Department of Quantitative Health Sciences, Cleveland Clinic, Cleveland, USA; 4 Division of Pulmonary and Critical Care Medicine, University of Washington, Washington, USA; 5 Pathology and Laboratory Medicine Institute, Cleveland Clinic, Cleveland, USA; 6 Pulmonary, Allergy, Critical Care, and Sleep Medicine, Minneapolis VA Health Care System, Minneapolis, MN USA; 7 Department of Inflammation and Immunity, Lerner Research Institute, Cleveland Clinic, Cleveland, USA; 8 Department of Internal Medicine, Division of Pulmonary Medicine, Medical University of South Carolina, Charleston, South Carolina USA; 9 Department of Internal Medicine, Division of Respiratory Medicine, Mayo Clinic, Rochester, USA; 10 Education Institute, Cleveland Clinic, Cleveland, USA; 11 9500 Euclid Avenue, Cleveland, OH 44195 USA

**Keywords:** Alpha-1 antitrypsin, COPD, Exacerbations, Healthcare utilizations

## Abstract

**Background:**

The best described endotype of COPD is alpha-1 antitrypsin (AAT) deficiency, due to a genetic abnormality in the *SERPINA1* gene. Common deficient PI variants are the Z and S variants. Homozygotes for the Z allele (PI*ZZ individuals) carry the genotype most commonly associated with severe AAT deficiency (AATD), but a highly prevalent endotype is the heterozygous state (PI*MZ individuals). The effect of PI*MZ status on exacerbations and health care utilization is unknown.

**Study design and methods:**

Cleveland electronic health record data was examined to compare healthcare utilization between PI*MZ and PI*MM individuals. Three outcomes were assessed: moderate COPD exacerbation (defined as short-term steroid prescription), any emergent care (defined as an express care, urgent care, or emergency department visit), and any hospitalization. Models were adjusted for age, sex, race, BMI, smoking status, comorbidity count, liver disease, zip code median income.

**Results:**

4,148 individuals had the PI*MM genotype and 308 PI*MZ. PI*MZ was associated with increased risk for moderate COPD exacerbations (HR [95% CI]: 1.66 [1.27, 2.17]) and hospitalizations (HR [95% CI]: 1.44 [1.19, 1.75]) compared to PI*MM. The risk of hospitalization was higher among PI*MZ individuals with AAT levels < 90 mg/dL (HR [95% CI]: 1.59 [1.14, 2.23]) but not in those with AAT levels > 90 mg/dL, as compared to PI*MM.

**Interpretation:**

Given the high prevalence, PI*MZ represents a COPD phenotype that is associated with worse outcomes, inviting additional investigation to identify predictive biomarkers of worse disease and treatable traits. Future prospective studies to better characterize the longitudinal course and healthcare utilization among individuals with a PI*MZ genotype.

**Supplementary Information:**

The online version contains supplementary material available at 10.1186/s12931-025-03322-6.

## Background

Chronic obstructive pulmonary disease (COPD) is the fourth leading cause of death worldwide [1]. Treatment modalities have remained relatively limited and there have been continued efforts to identify specific endotypes of COPD, particularly those amenable to specific therapy. The best described endotype remains alpha-1 antitrypsin (AAT) deficiency, which is a result of a genetic abnormality in the *SERPINA1* gene that encodes for AAT [2]. The M-type protease inhibitor (PI) is the most common and wild-type allele of AAT in the general population. Common deficient PI variants seen in patients with AATD include the Z and S variants; homozygotes for the Z allele, called PI*ZZ individuals, carry the genotype most commonly associated with severe AAT deficiency (AATD) and clinical disease; the PI*ZZ genotype accounts for 1–3% of individuals with COPD. An understudied, but highly prevalent (~ 3% prevalence in the United States [3] and affecting more than 35,000,000 globally [4]) potential endotype is the heterozygous state with one wild-type allele (M) and one deficient allele (Z), hereafter referred to as PI*MZ.

The AAT protein is a pleiotropic protein that is synthesized in the liver and functions to antagonize neutrophil elastase (NE). The pathogenesis of liver and lung disease associated with AATD differs. Liver disease is triggered by the intrahepatic accumulation of polymers of the Z AAT protein (“toxic gain of function”). Deficiency of AAT in the lung (“toxic loss of function”) predisposes to emphysema given unopposed NE proteolytic activity. Other clinical sequelae of AATD include bronchiectasis, panniculitis, and vasculitis, all of which can contribute to morbidity [8, 9, 10]. Beyond inhibiting NE, AAT also inhibits interleukin-8 (IL-8)-mediated neutrophil chemotaxis [5] and IL-8 has been shown to be increased in frequent exacerbators [6]. While PI*ZZ individuals with COPD have been shown to have frequent exacerbations [7], less is known about exacerbation frequency among PI*MZ individuals and how AAT levels may impact this. An analysis of the Copenhagen General Population Study showed that carrying a Z variant allele was associated with increased exacerbation frequency; because MZ, SZ, and ZZ genotypes were bundled in this study, conclusions regarding the risk conferred by the PI*MZ genotype could not be ascertained [8]. An increased serum AAT level in that study was associated with more frequent exacerbations but this was not assessed by genotype and likely reflects the role of AAT as an acute phase reactant [9]. Therefore, the role of the serum AAT level in the context of exacerbations remains unknown.

Epidemiologic studies have identified lower lung function and more emphysema among PI*MZ smokers [10, 11, 12]. However, less is known regarding the healthcare utilization patterns of PI*MZ individuals compared to their normal (alpha-1 antitrypsin-replete individuals [PI*MM genotype]) counterparts. Assessment of the SubPopulations and InteRmediate Outcome Measures in COPD Study (SPIROMICS) participants did not demonstrate an increased risk of exacerbation but included only 79 PI*MZ individuals and interaction by AAT level was not assessed [13]. Our prior assessment of 11 PI*MZ individuals in the Severe Asthma Research Program (SARP) study demonstrated increased asthma exacerbations in this small sample size [14]. Given the pleiotropic anti-inflammatory effects of the AAT protein, we hypothesized that PI*MZ individuals would have increased healthcare utilization and more frequent exacerbations compared to individuals without AATD, i.e., PI*MM, and that lower AAT serum level among PI*MZ individuals would be associated with higher healthcare utilization and more frequent exacerbations. To assess this hypothesis, existing data within the Cleveland Clinic Health System (CCHS) electronic health record (EHR) system were examined to compare healthcare utilization and mortality among individuals with these two genotypes.

## Methods

### Study cohort

Using the CCHS EHR (EPIC, Verona, WI), which includes billing information, helped characterize the population served by CCHS; [15] 70% of our patient population comes from our Northeast Ohio region [13], 20% comes from outside the 6-county area surrounding Cleveland, and 10% comes from more remote areas. The Cleveland Clinic can coordinate care outside its network by securely using EHR, sharing relevant patient information with external providers. Patients who underwent AAT genotype testing (using LightMix and LightSNiP technologies on the LightCycler 480 II) between 2016 and 2021 were identified and included in the analysis. Participants followed for fewer than 90 days within the CCHS system were excluded. Institutional review board approval was obtained from the Cleveland Clinic IRB (IRB #22–390) as minimal risk with waived consent.

### Study outcomes and group definitions

Extracted data include demographic characteristics and laboratory data, disease diagnoses (based on ICD-9 or ICD-10 diagnosis codes), medication prescription data, and healthcare utilization. A comorbidity count variable was created based on the ICD-diagnosis (Table E1) of: obstructive sleep apnea, diabetes, hypertension, obesity, gastroesophageal reflux disease, peptic ulcer disease, obesity, congestive heart failure, and peripheral arterial disease [16]. Race was categorized as white or black given that only one participant reported a different race. Income estimates were calculated as the ratio of zip code median income to federal poverty level and categorized as < 1.5, 1.5-2, and > 2 given implications of neighborhood residence on COPD morbidity [17]. 

In keeping with prior work [13], moderate COPD exacerbations were defined as receipt of an oral corticosteroid prescription for less than 28 days [15]. A composite outcome including express care visits, urgent care visits, and emergency department visits was created and denoted as “emergent care.” All-cause hospitalization was included as an outcome. Participants were followed from the date of AAT genotype testing for up to 12 months, until the occurrence of the event, or death, whichever came first.

A subgroup analysis was performed regarding individuals with preexisting COPD or respiratory disease, identified by having at least a six-month history of an inhaler prescription order at any time during follow-up. This analysis aimed to examine whether the associations observed were consistent among those with preexisting respiratory disease. Effect modification by smoking status was assessed in all participants and the preexisting respiratory disease subgroup. Lastly, to assess the effect of AAT levels on outcomes, participants were categorized into three groups based on AAT level and genotype. As 90 mg/dL is the lower limit of the normal reference range, the categories were: MM individuals with an AAT level > 90 mg/dL, MZ > 90 mg/dL and MZ < 90 mg/dL.

### Statistical analysis

Descriptive statistics were used to summarize characteristics in each group. Baseline characteristics were compared between the MM and MZ genotype by Wilcoxon rank-sum test for continuous variables or chi-squared for categorical variables as appropriate. Three outcomes were assessed: moderate COPD exacerbation (defined as a short-term steroid prescription), all-cause emergent care (defined as an express care visit, urgent care visit, or emergency department visit), and all-cause hospitalization. Univariate Kaplan-Meier curves were generated for all three outcomes with Greenwood confidence intervals. Time-to-event analyses with a Cox proportional hazard models were performed from the date of genotype testing to a maximum of 12 months, the outcome of interest, or death.

Models were adjusted for age at the time of genotype testing, sex, race (categorized as non-Hispanic white [NHW] or black), BMI (underweight [< 18.5], normal weight [18.5–24.9], overweight [25–30], or obese [> 30]), smoking status (former, never, or current), comorbidity count, liver disease diagnosis, ratio of zip code median income to federal poverty level (< 1.5, 1.5-2, and > 2).

A subgroup analysis was conducted among those with preexisting COPD or respiratory disease adjusted for the same covariates. Effect modification by smoking status (current/former versus never) was evaluated by including a multiplicative interaction between smoking status and genotype. To assess the influence of AAT level, participants were categorized into the three aforementioned groups. Cox proportional hazard models were then used to compare the MM individuals with high and low AAT levels to MM individuals adjusted for the same covariates. A sensitivity analysis was conducted in all models with: (a) inclusion of the outcome in the prior year as a covariate [18] and (b) pack-years as a covariate.

## Results

### Cohort characteristics

Of 4,456 participants with genotype testing who were followed for at least 90 days, 4,148 individuals with the MM genotype and 308 individuals with the MZ genotype were identified (Table 1). The median (interquartile) age was 56.8 (44.2, 65.8) years, 2,216 (49.7%) were female and 565 (13.5%) were Black. The groups were largely similar, although more MZ individuals were NHW, never smokers, and resided in higher income zip codes. Of all 4,456 participants, 622 (14.0%) experienced a moderate COPD exacerbation, 1,467 (32.7%) had an unscheduled emergent visit, and 1,487 (33.4%) had a hospitalization within one year of follow-up.

**Table 1:** Baseline characteristics

Characteristic	Overall, *N* = 4,456^1^	PI*MM, *N* = 4,148^1^	PI*MZ, *N* = 308^1^	*p*-value^2^
Age at Genotyping	56.8 (44.2, 65.8)	56.9 (43.9, 65.8)	56.0 (47.1, 64.6)	0.6
Gender				> 0.9
Male	2,239 (50.3%)	2,085 (50.3%)	154 (50.0%)	
Female	2,216 (49.7%)	2,062 (49.7%)	154 (50.0%)	
Missing, n	1	1	0	
Race				**< 0.001**
Non-Hispanic whites	3,564 (85.0%)	3,269 (84.0%)	295 (97.4%)	
Black	565 (13.5%)	558 (14.3%)	7 (2.3%)	
Other	64 (1.5%)	63 (1.6%)	1 (0.3%)	
Missing, n	261	257	4	
BMI				0.3
Normal18.5-25	1,184 (26.6%)	1,115 (26.9%)	69 (22.4%)	
Underweight < 18.5	177 (4.0%)	166 (4.0%)	11 (3.6%)	
Overweight 25-30	1,289 (28.9%)	1,189 (28.7%)	100 (32.5%)	
Obese > 30	1,806 (40.5%)	1,678 (40.5%)	128 (41.6%)	
Smoking Status				**0.002**
Never	1,993 (44.9%)	1,826 (44.2%)	167 (54.4%)	
Former	1,880 (42.4%)	1,769 (42.8%)	111 (36.2%)	
Current	565 (12.7%)	536 (13.0%)	29 (9.4%)	
Missing, n	18	17	1	
Inhaler prescription	2,489 (55.9%)	2,315 (55.8%)	174 (56.5%)	0.8
Median Income	61,853.0 (48,302.0, 77,366.0)	61,853.0 (47,559.0, 77,366.0)	63,007.0 (51,031.0, 80,205.0)	**0.010**
Missing, n	46	45	1	
Median Income Ratio				**0.006**
[0,1.5]	919 (20.8%)	877 (21.4%)	42 (13.7%)	
[1.5,2]	1,398 (31.7%)	1,290 (31.4%)	108 (35.2%)	
[2,7]	2,092 (47.4%)	1,935 (47.2%)	157 (51.1%)	
Missing, n	47	46	1	
Liver Disease	3,049 (68.4%)	2,855 (68.8%)	194 (63.0%)	**0.033**
CHF	815 (18.3%)	772 (18.6%)	43 (14.0%)	**0.042**
Antitrypsin level (mg/dL)	146.0 (126.0, 171.0)	150.0 (132.0, 173.8)	90.5 (80.0, 107.3)	**< 0.001**
Missing, n	2,434	2,322	112	
Eosinophil count	0.14 (0.08, 0.23)	0.14 (0.08, 0.23)	0.15 (0.09, 0.26)	0.2
Missing, n	418	396	22	
FEV1%, Pre-BD	74.0 (52.9, 91.0)	74.0 (52.1, 91.0)	75.5 (55.0, 90.6)	0.6
Missing, n	3,336	3,130	206	
FVC%, Pre-BD	83.8 (70.0, 98.0)	83.0 (69.3, 98.0)	87.5 (73.0, 101.0)	**0.049**
Missing, n	3,336	3,130	206	

^1^Median (IQR); n (%)

^2^Wilcoxon rank sum test; Pearson’s Chi-squared test; Fisher’s exact test

BD– Bronchodilator

### Increased exacerbations and hospitalizations among MZ compared to MM individuals

**Fig. 1:**
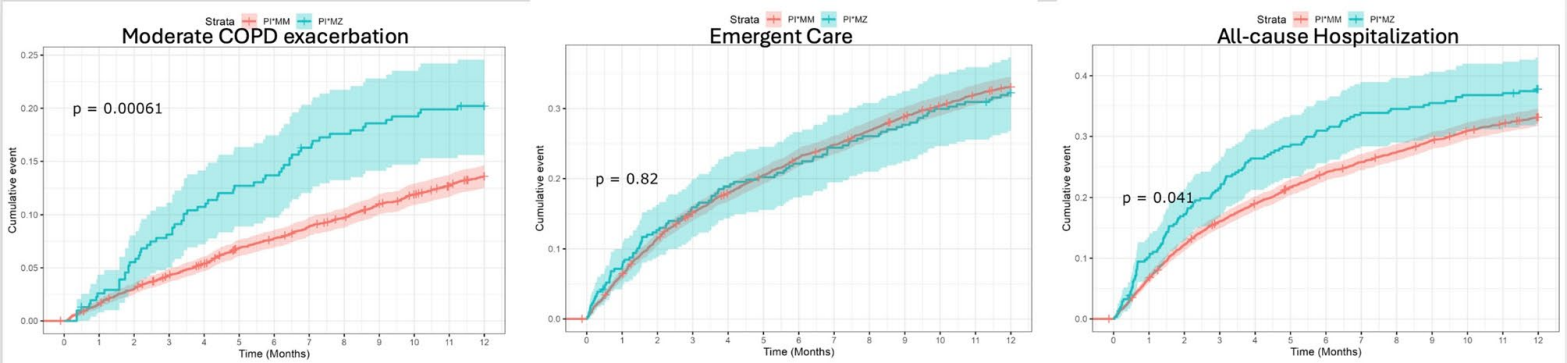
Kaplan-Meier curves comparing outcomes among PI*MZ and PI*MM individuals: (**a**) Moderate COPD exacerbation, (**b**) Any emergent care, and (**c**) All-cause hospitalizations

In univariate models, Cox regression analysis showed a higher hazard of experiencing a moderate COPD exacerbation among MZ individuals compared to MM individuals (HR 1.58, 95% CI: 1.21, 2.05; *p* < 0.01), no difference in emergent care (HR 0.98, 95% CI: 0.80, 1.20; *p* = 0.82), and a higher hazard of hospitalization among MZ compared to MM individuals (HR 1.22, 95% CI: 1.01, 1.47; *p* = 0.04) (Fig. 1).

In the adjusted models, Cox regression analysis showed a higher hazard of experiencing a moderate COPD exacerbation among MZ individuals compared to MM individuals (HR 1.44, 95% CI: 1.19, 1.75; *p* < 0.01). There was a higher hazard of emergent care (HR 1.23, 95% CI: 1.00, 1.51; *p* = 0.05), with borderline statistical significance. There was a higher hazard of hospitalization among MZ compared to MM individuals (HR 1.44, 95% CI: 1.19, 1.75; *p* < 0.01).

### Subgroup and sensitivity analysis

Of the total cohort of 4,456, 2,489 had a preexisting obstructive respiratory condition as defined by an inhaler prescription for at least six months. Similar to the whole cohort findings, a higher hazard for moderate COPD exacerbations (HR 1.59, 95% CI: 1.15, 2.18; *p* < 0.01) and hospitalizations (HR 1.52, 95% CI: 1.19, 1.93; *p* < 0.01) was observed in this subset. There was a directionally higher hazard for emergent care (HR 1.15, 95% CI: 0.90, 1.49; *p* = 0.27) that did not achieve statistical significance. There was no interaction by smoking status in either the all-participant group or the respiratory medication only group (Table E2).

Of the total cohort of 4,456, 2,022 had available AAT levels. These 2,022 individuals were compared to the 2,434 with missing AAT levels. The groups were similar except that those without levels were more likely to be never smokers and to have liver disease (Table E3). Of the 1,818 MM individuals with AAT levels, all were > 90 mg/dL. Of the 196 MZ individuals with available AAT levels, 101(52%) were > 90 mg/dL and 95 (48%) were < 90 mg/dL. The hazard of experiencing an exacerbation or emergent care was generally the same among MZ with normal or abnormal levels (Table 2). However, there was a higher hazard of a hospitalization among MZ individuals with an AAT level < 90 mg/dL.

**Table 2:** Hazard ratios for outcomes among PI*MZ versus PI*MM individuals

	All-cause Hospitalization		All-cause Emergent Care		Moderate COPD Exacerbation	
Sample	HR (95% CI)^1^	*p*-value	HR (95% CI)^1^	*p*-value	HR (95% CI)^1^	*p*-value
**All Patients**						
PI*MZ	1.44 (1.19, 1.75)	**< 0.001**	1.23 (1.00, 1.51)	*0.051*	1.66 (1.27, 2.17)	**< 0.001**
**Subgroup: Respiratory Medication**						
PI*MZ	1.52 (1.19, 1.93)	**< 0.001**	1.15 (0.90, 1.49)	0.27	1.59 (1.15, 2.18)	**< 0.001**
**Subgroup: AAT Level**						
PI*MZ normal	1.21 (0.86, 1.70)	0.27	1.29 (0.92, 1.80)	0.13	1.60 (1.02, 2.51)	**0.040**
PI*MZ abnormal	1.59 (1.14, 2.23)	**< 0.001**	1.15 (0.78, 1.71)	0.47	1.60 (1.00, 2.57)	**0.050**

^1^HR = Hazard Ratio, CI = Confidence Interval. Models were adjusted for age at the time of genotype testing, sex, race (categorized as non-Hispanic white [NHW] or black), BMI (underweight [< 18.5], normal weight [18.5–24.9], overweight [25–30], or obese [> 30]), smoking status (former, never, or current), comorbidity count, liver disease diagnosis, ratio of zip code median income to federal poverty level (< 1.5, 1.5-2, and > 2)

Sensitivity analysis adjusting for the occurrence of an outcome (e.g. moderate COPD exacerbation) in the prior year showed similar but attenuated results (Tables E4).

Pack-year data was available in 3,439 (82.9%) MM and 276 (89.6%) MZ participants. The median (interquartile) pack-years was 20 (10, 40) for ever-smokers in both groups. Sensitivity analysis adjusting for the pack-years demonstrated similar results (Table E5).

## Discussion

In this comprehensive evaluation of real-world data, individuals with PI*MZ experienced higher risks of moderate COPD exacerbations and all-cause hospitalizations compared to individuals with PI*MM. Notably, those with PI*MZ levels and AAT levels < 90 mg/mL exhibited marked higher risk of hospitalizations. Our results highlight the clinical importance of identifying PI*MZ status and emphasize the need for targeted interventions and close monitoring of this at-risk population, especially those with lower AAT levels.

There were differences at baseline between the MZ and MM groups. Notably, there were a higher number of never smokers among MZ group and the MZ group resided in higher income zip codes. These differences would likely bias toward fewer exacerbations and hospitalizations among this group. The MZ group also had a higher number of NHW (97.4%) compared to black individuals (2.3%). Overall, 8.3% of the NHW tested were found to be MZ, whereas only 1.2% of blacks tested were MZ. The paucity of PI*MZ who were black is concordant with the observation that the Z allele is more prevalent among those of European lineage, although black individuals may have higher percentage of other rare *SERPINA1* alleles [19]. 

These findings both extend and are discordant with those of a prior study in which data from several studies (COPDGene, LTRC, and ECLIPSE) were combined; in that experience, the odds ratio for COPD exacerbations was similar among the two compared groups, though the proportion of current smokers was much higher among PI*MM individuals (39% current smokers PI*MM versus 22% in PI*MZ across the three cohorts) [20]. Notably, prescription and hospitalization rates were not available in that study and exacerbations were self-reported. A directionally higher but not significant incidence rate of exacerbations was observed among the PI*MZ individuals in the SPIROMICS cohort, but only 79 PI*MZ individuals were included which may have underpowered the analysis [13]. 

It is perhaps surprising that no differences in FEV1% predicted were observed between PI*MM and PI*MZ individuals in our cohort, though there was also a large degree of missingness for lung function. Furthermore, only pre-bronchodilator lung function was assessed, which limited our ability to assess those with diagnosed COPD. Still, to adjudicate the impact among those with COPD or respiratory disease, a sensitivity analysis was conducted among those on inhaler medication; this demonstrated similar results. Given that the pulmonary risk conferred by AATD is greater in ever smokers [19, 21], the impact of smoking status was examined; no interaction was found. The high degree of missingness of data on pack-years of smoking precluded a more detailed analysis by smoking exposure. Though non-evaluable in our data, it is possible that the absence of a smoking effect on pulmonary risk relates to minimal pack-years among both current and former smokers in our cohort.

The increased risk of hospitalization among PI*MZ individuals with a serum AAT level < 90 mg/dL is noteworthy. This novel finding is noteworthy in the context that pulmonary outcomes are heterogeneous among different PI*MZ subgroups [19, 20] and, in keeping with dosing studies for augmentation therapy [21], suggests that serum AAT level may influence outcomes in PI*MZ individuals. A discordant finding in our study is that the frequency of moderate COPD exacerbations did not differ among PI*MZ individuals with serum AAT levels above vs. below 90 mg/dl.

Several limitations of the current study warrant discussion. First, usual limitations associated with data captured from the EHR apply. For example, compounding the unavailability of systematically captured data on pack-years of smoking, steroid prescriptions and hospitalizations outside of the CCHS were not accounted for in our study. Though including only individuals with longitudinal outpatient follow-up at the CCHS for at least 90 days may mitigate this potential confounder, bias related to steroid prescriptions and healthcare visits outside CCHS may still persist. Whether the steroid prescriptions were for a COPD exacerbation or potentially an asthma [14] or bronchiectasis [21] exacerbation cannot be adjudicated. Furthermore, indications for steroid prescriptions that were found may regard non-pulmonary issues. We did not include antibiotics to minimize this possibility as antibiotics are more likely to be prescribed for non-pulmonary causes. A second limitation is that this was a single center experience and requires replication in prospective studies of better characterized AATD individuals (e.g., with pack-years of smoking exposure available). Also, the use of all-cause hospitalization and all-cause emergent care may obscure the connection between AATD and the reason for hospitalization. Finally, the results could be confounded by the availability of serum AAT levels in only a subset and that inflammatory markers– which are associated with serum AAT levels as an acute phase reactant– were unavailable. Offsetting these limitations are that these data reflect real-world experience in a large number of PI*MZ individuals [16].

In summary, the study found increased all-cause hospitalization and moderate COPD exacerbations among PI*MZ individuals compared to PI*MM individuals in a real-world setting. This underscores the relevance of continued efforts of enhancing AAT genotype testing as fewer than 15% of patients in whom AATD testing is indicated are actually tested [22]. Future prospective studies are needed to better characterize the longitudinal course, healthcare utilization, and biomarkers of more severe disease among heterozygotes, especially because PI*MZ individuals comprise ~ 3% of the U.S. population and many more people globally.

## Electronic supplementary material

Below is the link to the electronic supplementary material.

Supplementary Material 1

## Data Availability

No datasets were generated or analysed during the current study.

## Declarations

### Prior presentation

Preliminary results of this work were presented at the American Thoracic Society Conference in May, 2023.

## References

[1] Kochanek KD, Murphy SL, Xu J, Arias E. Mortality in the united states, 2016. NCHS Data Brief. 2017;(293):1–8. 29319473

[2] Tejwani V, Stoller JK. The spectrum of clinical sequelae associated with alpha-1 antitrypsin deficiency. Ther Adv Chronic Dis. 2021;12suppl:2040622321995691. 10.1177/2040622321995691. 10.1177/2040622321995691 PMC8367210 34408829

[3] de Serres FJ, Blanco I. Prevalence of α1-antitrypsin deficiency alleles PI*S and PI*Z worldwide and effective screening for each of the five phenotypic classes PI*MS, PI*MZ, PI*SS, PI*SZ, and PI*ZZ: a comprehensive review. Ther Adv Respir Dis. 2012;6(5):277–95. 10.1177/1753465812457113. 22933512 10.1177/1753465812457113

[4] Martinez-González C, Blanco I, Diego I, Bueno P, Miravitlles M. Estimated prevalence and number of PiMZ genotypes of Alpha-1 antitrypsin in Seventy-Four countries worldwide. Int J Chron Obstruct Pulmon Dis. 16:2617–30. 10.2147/COPD.S327803 10.2147/COPD.S327803 PMC8455519 34556982

[5] Bergin DA, Reeves EP, Meleady P, et al. α-1 antitrypsin regulates human neutrophil chemotaxis induced by soluble immune complexes and IL-8. J Clin Invest. 2010;120(12):4236–50. 10.1172/JCI41196. 21060150 10.1172/JCI41196 PMC2993580

[6] Tumkaya M, Atis S, Ozge C, Delialioglu N, Polat G, Kanik A. Relationship between airway colonization, inflammation and exacerbation frequency in COPD. Respir Med. 2007;101(4):729–37. 10.1016/j.rmed.2006.08.020. 17002892 10.1016/j.rmed.2006.08.020

[7] Smith DJ, Ellis PR, Turner AM. Exacerbations of lung disease in Alpha-1 antitrypsin deficiency. Chronic Obstr Pulm Dis Miami Fla. 2021;8(1):162–76. 10.15326/jcopdf.2020.0173. 10.15326/jcopdf.2020.0173 PMC8047608 33238089

[8] Ingebrigtsen TS, Marott JL, Rode L, Vestbo J, Lange P, Nordestgaard BG. Fibrinogen and α1-antitrypsin in COPD exacerbations. Thorax. 2015;70(11):1014–21. 10.1136/thoraxjnl-2015-207561. 26304913 10.1136/thoraxjnl-2015-207561

[9] Sanders CL, Ponte A, Kueppers F. The effects of inflammation on alpha 1 antitrypsin levels in a National screening cohort. COPD. 2018;15(1):10–6. 10.1080/15412555.2017.1401600. 29381093 10.1080/15412555.2017.1401600

[10] Hersh CP, Dahl M, Ly NP, Berkey CS, Nordestgaard BG, Silverman EK. Chronic obstructive pulmonary disease in alpha1-antitrypsin PI MZ heterozygotes: a meta-analysis. Thorax. 2004;59(10):843–9. 10.1136/thx.2004.022541. 15454649 10.1136/thx.2004.022541 PMC1746834

[11] Dahl M, Tybjaerg-Hansen A, Lange P, Vestbo J, Nordestgaard BG. Change in lung function and morbidity from chronic obstructive pulmonary disease in alpha1-antitrypsin MZ heterozygotes: A longitudinal study of the general population. Ann Intern Med. 2002;136(4):270–9. 10.7326/0003-4819-136-4-200202190-00006. 11848724 10.7326/0003-4819-136-4-200202190-00006

[12] Al Ashry HS, Strange C. COPD in individuals with the PiMZ alpha-1 antitrypsin genotype. Eur Respir Rev Off J Eur Respir Soc. 2017;26(146):170068. 10.1183/16000617.0068-2017. 10.1183/16000617.0068-2017 PMC9488576 29070580

[13] Barjaktarevic I, Hixson R, Zhuang Z, et al. Longitudinal outcomes in Pi*MZ Alpha-1 antitrypsin deficient individuals with tobacco smoking history from the SPIROMICS cohort. Ann Am Thorac Soc Published Online March. 2025;11. 10.1513/AnnalsATS.202411-1209OC. 10.1513/AnnalsATS.202411-1209OC PMC12254153 40068143

[14] Ortega VE, Tejwani V, Shrivastav AK et al. α1-Antitrypsin gene variation associates with asthma exacerbations and related health care utilization. J Allergy Clin Immunol Pract. Published online February 13, 2025:S2213-2198(25)00164-3. 10.1016/j.jaip.2025.01.039 10.1016/j.jaip.2025.01.039 PMC13285010 39954727

[15] Wu CP, Sleiman J, Fakhry B, et al. Novel machine learning identifies 5 asthma phenotypes using cluster analysis of Real-World data. J Allergy Clin Immunol Pract. 2024;12(8):2084–e20914. 10.1016/j.jaip.2024.04.035. 38685479 10.1016/j.jaip.2024.04.035 PMC11340628

[16] Putcha N, Puhan MA, Drummond MB, et al. A simplified score to quantify comorbidity in COPD. PLoS ONE. 2014;9(12):e114438. 10.1371/journal.pone.0114438. 25514500 10.1371/journal.pone.0114438 PMC4267736

[17] Raju S, Keet CA, Paulin LM, et al. Rural residence and poverty are independent risk factors for chronic obstructive pulmonary disease in the united States. Am J Respir Crit Care Med. 2019;199(8):961–9. 10.1164/rccm.201807-1374OC. 30384774 10.1164/rccm.201807-1374OC PMC6467317

[18] Hurst JR, Vestbo J, Anzueto A, et al. Susceptibility to exacerbation in chronic obstructive pulmonary disease. N Engl J Med. 2010;363(12):1128–38. 10.1056/NEJMoa0909883. 20843247 10.1056/NEJMoa0909883

[19] Ortega VE, Li X, O’Neal WK, et al. The effects of rare SERPINA1 variants on lung function and emphysema in SPIROMICS. Am J Respir Crit Care Med. 2020;201(5):540–54. 10.1164/rccm.201904-0769OC. 31661293 10.1164/rccm.201904-0769OC PMC7047460

[20] Ghosh AJ, Hobbs BD, Moll M, et al. Alpha-1 antitrypsin MZ heterozygosity is an endotype of chronic obstructive pulmonary disease. Am J Respir Crit Care Med. 2022;205(3):313–23. 10.1164/rccm.202106-1404OC. 34762809 10.1164/rccm.202106-1404OC PMC8886988

[21] Izquierdo M, Marion CR, Genese F, et al. Impact of bronchiectasis on COPD severity and Alpha-1 antitrypsin deficiency as a risk factor in individuals with a heavy smoking history. *Chronic obstr pulm dis Miami Fla*. Published Online May. 2023;16. 10.15326/jcopdf.2022.0388. 10.15326/jcopdf.2023.0388 PMC10484491 37199731

[22] Stoller JK. Detecting Alpha-1 antitrypsin deficiency: current state, impediments, opportunities, and future directions. Ann Am Thorac Soc. 2024;23. 10.1513/AnnalsATS.202406-600FR. Published online September. 10.1513/AnnalsATS.202406-600FR 39311761

