# Supplementary material for: Increased exacerbations and hospitalizations among PI*MZ compared to PI*MM individuals: an electronic health record analysis
Source: Respir Res. 2025 Jul 11;26:243. doi: 10.1186/s12931-025-03322-6 (PMC12247371; doi:10.1186/s12931-025-03322-6)
Supplement: Supplementary file 1 — Supplementary Material 1 [file 12931_2025_3322_MOESM1_ESM.docx]

**Table E1. Comorbid conditions: ICD 9 CM and ICD 10 CM codes**

| Comorbidity | ICD-9 | ICD-10 |
| --- | --- | --- |
| Obstructive sleep apnea | 780.57, 786.03, 327.23 | G47.30, R06.81, G4.33 |
| Diabetes | 250.00-250.03, 250.10-250.93 | E10.9, E11.9, E11.0 - E11.8, E10.0 - E10.8 |
| Hypertension | 401.1, 401.9, 642.00-642.04, 401.0, 402.00 - 402.90, 403.00 - 403.90, 404.00, 404.10, 404.90, 405.01- 405.99, 642.10 -462.24, 642.70 - 642.94 | I10, O10.019, O10.919, O10.011 - O10.013, O10.02, O10.911 - O10.913, O10.92, O10.03, O10.93, I11.9, I12.9, I13.10, I15.0, I15.8, O10.419, O10.13, O10.23, O10.33, O11.9, O16.1, O16.2, O16.3 |
| Gastroesophageal reflux disease | 530.11, 530.81 | K21.0, K21.9 |
| Peptic ulcer disease | 531.41 - 531.91, 532.41 - 532.91, 533 - 533.91, 534.41 - 534.90, V12.71 | K25.3, K25.4 - K25.9, K26.3 - K26.9, K27.3 -K27.9, K28.3 - K28.9, Z87.11 |
| Congestive heart failure | 398.91, 402.01, 402.11, 402.91, 404.01, 404.11, 404.91, 428.0- 428.9 | I09.81, I11.0, I13.0, I50.x, I50.xx. |
| Peripheral arterial disease | 440.0-440.9, 441.00-441.9, 442.0- 442.9, 443.1-443.9, 447.1, 557.1, 557.9, V43.4 | I70.0, I70.1, I70.209, I70.219, I70.229, I70.25, I70.269, I70.299- I70.599, I70.8, I70.90-I70.92, I71.00-I71.03, I71.1- I71.9, I72.0- I72.4, I72.8, I72.9, I73.1, I77.71- I77.74, I77.79, I79.8, I73.81, I73.89, I73.9, I77.1, K55.1, K55.9, Z95.828 |
| Liver disease | 070.22, 070.23, 070.32, 070.33, 070.44, 070.54, 456.0, 456.1, 456.20, 456.21, 571.0, 571.2, 571.3, 571.40- 571.49, 571.5, 571.6, 571.8, 571.9, 572.3, 572.8, V42.7 | B18.0, B18.1, B18.2, B18.8, B18.9, I85.0, I85.00, I85.01, I85.1, I85.10, I85.11, 45.21, K70.0, K70.1, K70.10, K70.11, K70.2, K70.3, K70.30, K70.31, K70.4 - K70.41, K70.9, K72.0 - K72.9, K72.90, K72.91, K73.0 - K73.9, K74.0 - K74.6, K74.60, K74.69, K75.2 - K75.8, K75.81, K75.89, K75.9, K76.0 - K76.8, K76.81, K76.89, K76.9, Z94.4 |

**Table E2. Interaction Term by Smoking Status (Ever Smokers versus Never Smokers)**

| **Sample** | **p-value for interaction*** |
| --- | --- |
| **All Patients** |  |
| Hospitalization | 0.53 |
| Emergent care | 0.43 |
| Moderate COPD exacerbation | 0.55 |
| **Respiratory medication subgroup** |  |
| Hospitalization | 0.34 |
| Emergent care | 0.94 |
| Moderate COPD exacerbation | 0.80 |
| *Multiplicative interaction for genotype and smoking status | |

##

**Table E3. Baseline Characteristics of Those with and without an AAT Level**

| **Characteristic** | **AAT Available**, N = 2,022^1^ | **AAT Missing**, N = 2,434^1^ | **p-value**^2^ |
| --- | --- | --- | --- |
| Age at genotyping | 57 (44, 66) | 57 (44, 66) | 0.5 |
| Gender |  |  | 0.7 |
| Male | 1,010 (45%) | 1,229 (55%) |  |
| Female | 1,011 (46%) | 1,205 (54%) |  |
| Missing, n | 1 | 0 |  |
| Race |  |  | 0.5 |
| Non-Hispanic white | 1,650 (46%) | 1,914 (54%) |  |
| Black | 250 (44%) | 315 (56%) |  |
| Other | 25 (39%) | 39 (61%) |  |
| Missing, n | 96 | 165 |  |
| BMI |  |  | 0.4 |
| Underweight<18.5 | 89 (50%) | 88 (50%) |  |
| Normal18.5-25 | 546 (46%) | 638 (54%) |  |
| Overweight25-30 | 590 (46%) | 699 (54%) |  |
| Obese>30 | 797 (44%) | 1,009 (56%) |  |
| Smoking Status |  |  | **0.016** |
| Never | 863 (43%) | 1,130 (57%) |  |
| Former | 900 (48%) | 980 (52%) |  |
| Current | 253 (45%) | 312 (55%) |  |
| Missing, n | 6 | 12 |  |
| Inhaler prescription |  |  | 0.9 |
| No | 895 (46%) | 1,072 (54%) |  |
| Yes | 1,127 (45%) | 1,362 (55%) |  |
| Median Income | 61,321 (48,302, 77,137) | 62,023 (47,559, 77,366) | 0.5 |
| Missing, n | 23 | 23 |  |
| Median Income Ratio |  |  | 0.2 |
| (0,1.5] | 410 (45%) | 509 (55%) |  |
| (1.5,2] | 663 (47%) | 735 (53%) |  |
| (2,7] | 925 (44%) | 1,167 (56%) |  |
| Missing, n | 24 | 23 |  |
| Liver Disease |  |  | **<0.001** |
| 0 | 697 (50%) | 710 (50%) |  |
| 1 | 1,325 (43%) | 1,724 (57%) |  |
| CHF |  |  | 0.4 |
| 0 | 1,664 (46%) | 1,977 (54%) |  |
| 1 | 358 (44%) | 457 (56%) |  |
| Antitrypsin level (mg/dL) | 146 (126, 171) | NA (NA, NA) |  |
| Missing, n | 0 | 2,434 |  |
| Eosinophil count | 0.14 (0.08, 0.23) | 0.14 (0.08, 0.23) | 0.9 |
| Missing, n | 186 | 232 |  |
| FEV1%, Pre-BD | 71 (46, 91) | 78 (60, 91) | **<0.001** |
| Missing, n | 1,323 | 2,013 |  |
| FVC%, Pre-BD | 83 (69, 99) | 84 (73, 97) | 0.2 |
| Missing, n | 1,323 | 2,013 |  |
| ^1^Median (IQR); n (%) | | | |
| ^2^Wilcoxon rank sum test; Pearson's Chi-squared test; Fisher's exact te | | | |

**Table E4. Hazard Ratios for Outcomes Among PI*MZ versus PI*MM Individuals adjusting for outcome in prior year**

|  | **All-cause Hospitalization** | | **All-cause Emergent Care** | | **Moderate COPD Exacerbation** | |
| --- | --- | --- | --- | --- | --- | --- |
| **Sample** | **HR** **(95% CI)**^1^ | **p-value** | **HR** **(95% CI)**^1^ | **p-value** | **HR** **(95% CI)**^1^ | **p-value** |
| **All Patients** | | | | | | |
| PI*MZ | 1.40 (1.15, 1.70) | **<0.001** | 1.19 (0.97, 1.47) | *0.09* | 1.65 (1.26, 2.16) | **<0.001** |
| **Subgroup: Respiratory Medication** | | | | | | |
| PI*MZ | 1.47 (1.16, 1.88) | **0.002** | 1.12 (0.87, 1.44) | 0.39 | 1.58 (1.15, 2.17) | **0.005** |
| **Subgroup: AAT Level** | | | | | | |
| PI*MZ normal | 1.06 (0.75, 1.50) | 0.73 | 1.23 (0.88, 1.73) | 0.23 | 1.59 (1.00, 2.52) | *0.05* |
| PI*MZ abnormal | 1.35 (0.96, 1.91) | *0.09* | 1.14 (0.77, 1.70) | 0.52 | 1.58 (0.97, 2.56) | *0.07* |
| ^1^HR = Hazard Ratio, CI = Confidence Interval. Models were adjusted for age at the time of genotype testing, sex, race (categorized as non-Hispanic white [NHW] or black), BMI (underweight [<18.5], normal weight [18.5-24.9], overweight [25-30], or obese [>30]), smoking status (former, never, or current), comorbidity count, liver disease diagnosis, ratio of zip code median income to federal poverty level (<1.5, 1.5-2, and >2), and occurrence of outcome in the year prior to genotype testing. | | | | | | |

**Table E5. Hazard Ratios for Outcomes Among PI*MZ versus PI*MM Individuals adjusting for pack years**

|  | **All-cause Hospitalization** | | **All-cause Emergent Care** | | **Moderate COPD Exacerbation** | |  |
| --- | --- | --- | --- | --- | --- | --- | --- |
| **Sample** | **HR** **(95% CI)**^1^ | **p-value** | **HR** **(95% CI)**^1^ | **p-value** | **HR** **(95% CI)**^1^ | **p-value** |  |
| **All Patients** | | | | | | |  |
| PI*MZ | 1.42 (1.16, 1.74) | **<0.001** | 1.26 (1.01, 1.56) | **0.04** | 1.72 (1.30, 2.28) | **<0.001** |  |
| ^1^HR = Hazard Ratio, CI = Confidence Interval. Models were adjusted for age at the time of genotype testing, sex, race (categorized as non-Hispanic white [NHW] or black), BMI (underweight [<18.5], normal weight [18.5-24.9], overweight [25-30], or obese [>30]), smoking status (former, never, or current), comorbidity count, liver disease diagnosis, ratio of zip code median income to federal poverty level (<1.5, 1.5-2, and >2), pack-years, and occurrence of outcome in the year prior to genotype testing. | | | | | | |  |
